# Measuring inequality in the distribution of health human resources using the Hirschman-Herfindahl index: a case study of Qazvin Province

**DOI:** 10.1186/s12913-022-08518-w

**Published:** 2022-09-14

**Authors:** Asghar Nasiri, Hasan Yusefzadeh, Mohammad Amerzadeh, Saeideh Moosavi, Rohollah Kalhor

**Affiliations:** 1 Student Research Committee, School of Public Health, Qazvin University of Medical Sciences, Qazvin, Iran; 2 Department of Health Management and Economics, School of Public Health, Urmia University of Medical Sciences, Urmia, Iran; 3 Social Determinants of Health Research Center, Research Institute for Prevention of Non-Communicable Diseases, Qazvin University of Medical Sciences, Qazvin, Iran; 4 Social Determinants of Health Research Center, Research Institute for Prevention of Non-Communicable Diseases, Qazvin University of Medical Sciences, Qazvin, Iran

**Keywords:** Health Human Resources, Herfindahl He-Shaman Index, Health Transformation Plan

## Abstract

**Background:**

Achieving equity in the distribution of health services is one major goal in the health system. This study aimed to determine equality in distributing health human resources (physicians) in the Qazvin University of Medical Sciences before and after the Health Transformation Plan (HTP) based on the Hirschman-Herfindahl index (HHI).

**Methods:**

In this descriptive-analytical study, the statistical population was general practitioners (GPs), specialists and subspecialists in the Qazvin University of Medical Sciences from 2011-to 2017. We extracted demographic statistics of the cities from the Statistical Center of Iran. Physicians’ statistics were obtained from the Curative Affairs Deputy at the Qazvin University of Medical Sciences. We assessed inequality using the HHI.

**Results:**

The highest number of GPs was in 2014, and specialists and subspecialists were in 2017. The lowest number of GPs, specialists, and subspecialists were in 2016, 2011 and 2015. The HHI for GPs in 2011–2017 was between 4300 and 5200. The lowest concentration for specialists before the HTP plan was the cardiologist with 3300, and after the HTP, the internal specialist with 3900. Also, the numerical value of this index for all subspecialty physicians after the HTP was 10,000, the highest level of concentration.

**Conclusion:**

The values obtained from the HHI index indicate the high concentration and disproportionate and inequitable distribution of human resources in the health sector in this province. The number of specialists in some cities is still much less than acceptable, and some cities even have shortcomings in the critical specialists.

## Background

Effective resource allocation, the effective participation of local, national and international communities at all levels, equity and accessibility to health services, and high-quality services are essential principles in providing, maintaining and promoting human health. [1].

One of the most important and valuable sources of health is the healthcare workforce. Health systems heavily depend on a sufficient and skilled workforce to provide high-quality services [2]. Most times, improper distribution of these resources can lead to a loss of health resources and impose additional costs on patients [3]. Equity in the health sector and the elimination of inequality in the distribution of health sector resources are among the significant concerns of health systems globally. Inequality in the distribution of human resources in the health sector has been a constant concern worldwide [4]. The results of research in the United States show that the availability and accessibility of health services causes a 22.9% reduction in mortality in the community. An average of 5 years of life expectancy has also increased with improving medical conditions in people [5]. The unequal distribution of physicians is increasingly more significant than in other human resources in health. It is a reality seen almost all over the world [6, 7]. Guinea, Indonesia and Paraguay have similar workforces, but there are vast differences in coverage. Ghaderi et al. showed that according to the Hirschman-Herfindahl index (HHI), inequality in the distribution of GPs in Sistan and Baluchestan, a Province in soth of Iran, in 2020 has decreased. However, inequality in the distribution of specialists remains at a high level [8]. Sugawara et al. studied psychiatrists' distribution for patients having mental illness via a utilization-based approach. They found that the mean number of psychiatrists per patient for patients having schizophrenia, mood disorders, vascular dementia, and Alzheimer’s disease in 2025, 2035, and 2045 was significantly lower than in 2015. For these diseases, both the Gini coefficients (GC) and HHI will increase until 2045 [9]. According to various studies in different countries, most physicians are concentrated in specific geographical areas, while other regions face a shortage of physicians [10]. They are not specific to developing countries [11, 12]. Therefore, adopting policies to increase health sector resources and reduce inequality in the distribution and allocation of these resources in different regions to establish equity in the distribution of human resources is necessary [11].

Providing human health resources and more access to them has a positive effect on the level of community health [13] but distributing mechanism of these resources is also very important [14]. Therefore, increasing resources in the health sector will not reduce inequality in the distribution of resources and better health [15]. Investigating the inequality of health resources is essential for establishing equity and reducing inequality. Therefore, it is crucial for health planners to study the inequality of health resources, especially human resources such as physicians, and how it is redistributed in different regions [16, 17].

At the beginning of 2014, the Ministry of Health and Medical Education (MoHME) of Iran designed and implemented a noticeable change in the country's health system through the Health Transformation Plan (HTP). It has eight service dimensions. Among the existing dimensions, several ones precisely follow the promoting equity and access to health resources: 1- ensuring fair and equitable distribution of physicians and subspecialists throughout the country 2- improving hoteling and renovation in the public sector [18]. In the beginning, the HTP primarily focused on treatment services. In August 2014, the HTP started focusing on primary health care services. In the next stage, the plan began in September 2014 with designing the new book: The Relative Value of Health Services. The performance-based payment system was implemented for other hospital staff working in January 2015 [18]. HTP, to some extent, resolved several urgent challenges, including the high rise of out-of-pocket and improved some health system's functions, including financing the health sector. It could also provide risk protection to some extent for the population [19].

Considering the implementation of the HTP since 2014 in the entire country, one of the essential principles of this plan is to promote equity and access to health resources [18]. Therefore, this study aimed to evaluate this plan by comparing the distribution of GPs, specialists and subspecialists working in health care networks affiliated with the Qazvin University of Medical Sciences before and after implementing the HTP using the HHI.

## Methods

This descriptive-analytical study was conducted to determine the equality in the distribution of human health resources at the Qazvin University of Medical Sciences before and after the HTP based on the HHI. The study population comprised subspecialties, specialists and GPs working at the Qazvin University of Medical Sciences from 2011 to 2017. According to the checklist designed, the number of physicians working in health care networks was obtained for each city from 2011 to 2017. It includes three parts: the study year, the population by each year, and the number of physicians (GPs, specialists with 26 orientations and subspecialists with 24 orientations). The sample studied in 2011 to 2017 were 542, 534, 545, 559, 521, 521, 395 GPs, 289, 297, 318, 311, 301, 397, 456 specialists, and 29, 28, 29, 28, 28, 39, 64 subspecialists. We collected data through the Statistical Center of Iran for the demographic information of each city. Since the population of cities and provinces is conducted every five years, only the population statistics of the target cities for 2011 and 2016 were available in statistical records. Therefore, the combined method of the Statistics Center of Iran was used to calculate the population of the cities. In this method, the population for the next year is equal to the number of the population last year, plus the number of births and net immigrants minus the number of deaths. Inclusion criteria included GPs, specialists, and subspecialties employed or licensed between 2011 and 2017 in the province's cities. Exclusion criteria also included GPs, specialists and subspecialists working in social security hospitals and the armed forces.

It was necessary to review and match the collected data to ensure accuracy. It was conducted by accessing the demographic information available in the University of Medical Sciences and Iran Medical Council, and the relevant gaps were corrected with the necessary follow-up. After ensuring the accuracy of the collected data, we performed calculations related to the HHI using a program designed in Excel software. This index is used to measure the share and concentration of firms in related industries. It is defined as the sum of the second power of each firm's share of the total industry output, and according to the use of information of all firms, each firm, based on the size of its share in the industry is given weight [20]. HHI is one index for measuring the concentration of market resources. Considering that this index pays attention to all points on the concentration curve and uses the information available throughout it, it is an important index for measuring concentration and inequality in the distribution of resources. This index is also used to examine the concentration on the health system. Based on HHI values of 0 to 1500 is not concentrated, 1500- to 2500 is moderately concentrated, and 2500 to 10 000 is concentrated [21, 22]. The collected data was entered into the HHI formula. As in the formula of this index, n is the number of cities in Qazvin Province, S is the share of each city of GPs, specialists and subspecialists, calculated after entering the information in Excel software according to the following formula.

$$HHI= \sum_{i=1}^{n}{\left(\frac{{X}_{i}}{X}\right)}^{2}$$

Xi is the number of variables by city and X is the number of the same variable in the whole province.

$$HHI=\sum_{i=1}^{n}{{S}_{i}}^{2}$$

## Results

The highest number of GPS before and after the HTP was in Qazvin city (389 people). The lowest number was in Avaj (1 person). After the implementation of the HTP, the number of infectious disease specialists (28), gynecologists (22), anesthesiologists (16), neurologists (8), radiologists (5) and pathologists (7), otolaryngologists (6) and emergency medicine specialists (*n* = 5) increased. A downward trend was observed in pediatric infectious diseases subspecialist (*n* = 1) and pediatric surgery (*n* = 1).

Since the HHI shows the sum of the squares of each city’s share of health resources, the lower the HHI is less than 1000, the lower the concentration of resources in the cities of the province and vice versa. (Table 1).

**Table 1:** HHI of GPs in Qazvin Province by year

Year	2011	2012	2013	2014	2015	2016	2017
City							
Qazvin	4900	5264/93	4804/44	4673/84	4419/37	5006/23	3987/28
Buin Zahra	73/02	26/58	42/22	37/83	51/21	14/23	24/14
Alborz	36	41/54	49/55	49/41	59/61	54/14	91/82
Abeik	29/75	24/72	24/70	22/51	23/97	25/95	24/14
Takestan	69/95	84/78	102/17	138/65	136/33	123/92	210/14
Avaj	0/033	0	0/032	0/030	0/035	0/03	0
HHI Index	5108/87	5445/33	5028/47	4925/39	4694/08	5227/41	4346/23

There is a high concentration in the number of GPs in the cities of Qazvin Province. The distribution of GPs, according to the population of cities, is unbalanced and unequal among different regions of the province. During this period, inequality in the distribution of GPs has decreased compared to the years before the HTP (except for 2016, the value of the index has gradually decreased).

According to Table 2, there was a high concentration in all studied specialties. The distribution of these specialties was unbalanced and unequal among different regions of the province according to the population of cities. Inequality in the distribution of specialties in ENT, ophthalmology, dermatology, psychiatry, radiology and emergency medicine has increased compared to the years before the HTP. However, in some specialties, such as anesthesiology, urology, pediatrics, internal medicine and neurology, inequality in the distribution has gradually decreased compared to the years before the HTP.

**Table 2:** Total HHI of specialists by year from 2011 to 2017

Field	The total index value of Hirschman Herfindahl Specialists by year from 2011 to 2017						
	2011	2012	2013	2014	2015	2016	2017
Otolaryngologist	7600	8673/46	6885/81	8755/55	8755/55	7600	8231/29
Ophthalmologist	6622/22	6234 / 56	6955/01	5734/07	7854/67	7118/05	7460/31
Urologist	8024/69	10,000	10,000	8200	7812/5	6033/05	5702/47
Infectious disease specialist	6250	10,000	6250	6250	6250	5510/20	8692/03
Gynecologist	5555/55	6533 / 33	7148/76	7148/76	7249/527	6345/66	5876/92
Pediatrician	4567/47	4186/85	4109/13	5622/22	5505/35	4679/75	4747/47
Internist	4876/54	4259/25	3650	4404/43	4404/43	4600	3984/37
Neurologist	10,000	10,000	7812/5	10,000	10,000	10,000	10,000
Dermatologist	7812/5	10,000	6543/21	6543/21	8024/69	8024/69	8200
Orthopedist	5495/86	6818/18	7229 / 91	6550	6398/89	6157/02	4624
General Surgeon	8060/94	8950/61	5963/71	7962/96	8673/46	7152	7174/51
Anesthesiologist	8600/82	7792	7708/33	8073/72	8133/33	6585/36	6266/66
Psychiatrist	6800	6859/50	5029/58	5266/27	7024/79	5644/44	7414/96
Rehabilitation specialist	10,000	10,000	10,000	10,000	10,000	10,000	10,000
Radiologist	5454/54	6186/55	6352/04	7969/82	6862/245	7 107/18	6250
Pathologist	10,000	7551/02	6859/50	8200	8200	7037/03	5488
Cardiologist	4700	3308/12	3595/04	5328/79	5937/5	5650/88	4230/76
Sports Medicine specialist	-	-	-	10,000	-	10,000	10,000
Occupational Medicine specialist	5555 / 55	5555/55	5555/55	5555/55	6800	10,000	10,000
Nuclear Medicine specialist	10,000	10,000	10,000	10,000	10,000	10,000	10,000
Radiotherapist	10,000	10,000	10,000	10,000	10,000	10,000	10,000
Forensic	10,000	10,000	10,000	10,000	10,000	10,000	10,000
Neurologist	10,000	6800	4400	Radiotherapy	medicine	6600	5702/47
Social Medicine specialist	10,000	10,000	10,000	10,000	10,000	10,000	10,000
Traditional medicine specialist	-	-	10,000	10,000	10,000	10,000	10,000
Emergency medicine specialist	-	-	-	-	-	5200	8200

The focus on orthopedics, infectious diseases, neurosurgery, rehabilitation, nuclear medicine, radiotherapy, oncology, forensics, social medicine and traditional medicine has not changed and remains high compared to the years before the HTP.

According to Table 3, there was a high concentration in all studied specialties, and the distribution of these specialties was unbalanced and unequal among different regions. Some of the subspecialty fields after the HTP have had an unbalanced distribution and high concentration. The distribution of some fields such as reconstructive surgery, thoracic surgery, pediatric infection, rheumatology, nephrology, blood and oncology, gastroenterology, lung, pediatric glands, pediatrics, endocrinology, pediatric surgery, pediatric neurology and cardiovascular surgery, etc. have not changed after the HTP.

**Table 3:** HHI of all subspecialists in Qazvin province

**Field**	The value of the total Hirschman-Herfindahl index of all physicians 2011–2017						
	**2011**	**2012**	**2013**	**2014**	**2015**	**2016**	**2017**
Allergy and immunology	-	-	-	-	-	10,000	10,000
Neonatal	-	-	-	-	-	10,000	10,000
Reconstructive surgery	10,000	10,000	10,000	10,000	10,000	10,000	10,000
Thoracic surgery	10,000	10,000	10,000	10,000	10,000	10,000	10,000
Pediatric Infection	10,000	10,000	10,000	10,000	10,000	10,000	10,000
Rheumatology	10,000	10,000	10,000	10,000	10,000	10,000	10,000
Adult nephrology	10,000	10,000	10,000	10,000	10,000	10,000	10,000
Pediatric nephrology	-	-	-	-	-	10,000	10,000
Blood and oncology	10,000	10,000	10,000	10,000	10,000	10,000	10,000
Gastroenterology	10,000	10,000	10,000	10,000	10,000	10,000	10,000
Lung	10,000	10,000	10,000	10,000	10,000	10,000	10,000
Pediatric endocrinology	10,000	10,000	10,000	10,000	10,000	10,000	10,000
Pediatric	10,000	10,000	10,000	10,000	10,000	10,000	10,000
Pediatric surgery	Pediatric	glands	10,000	10,000	10,000	10,000	10,000
Endocrinology	10,000	10,000	10,000	10,000	10,000	10,000	10,000
Pediatric neurology	-	10,000	10,000	10,000	10,000	10,000	10,000
Cardiovascular surgery	10,000	-	10,000	10,000	10,000	10,000	10,000
Pediatric Psychiatry	-	-	10,000	10,000	10,000	-	10,000
Pediatric Blood and Oncology	-	-	-	10,000	10,000	-	10,000
Cardiovascular	-	-	-	-	-	10,000	10,000
Pediatric Gastroenterology	-	-	-	-	-	10,000	10,000
Vascular Surgery	-	-	-	-	-	-	10,000
Pediatric Heart	-	-	-	-	-	-	10,000
Pediatric Urology	-	-	-	-	-	-	10,000

## Discussion

This study aimed to evaluate this plan by comparing the distribution of GPs, specialists and subspecialists working in health care networks affiliated with the Qazvin University of Medical Sciences before and after implementing the HTP using the HHI. Our findings showed that the HTP has not been successful in the equitable distribution of human resources. Qazvin Province, and five other provinces, including Sistan and Baluchestan, Ardabil, Kohgiluyeh and Boyer-Ahmad and Hormozgan, have the lowest level of development in healthcare services [23]. Investigating the inequality of health resources is necessary to establish equality and reduce inequality. Therefore, it is crucial for healthcare managers to understand the inequality of health resources, especially human resources such as physicians, and how it is redistributed in different regions [24].

The values obtained from the HHI indicate the high concentration and disproportionate and inequitable distribution of human resources in the health sector in Qazvin province after implementing the HTP. There is also a high concentration of the number of GPs in the cities of Qazvin Province. The distribution of GPs is unbalanced and unequal among different regions of the province since HHI was 3987/28 in 2017 compared to a smaller number in other cities in Qazvin province. During this period, inequality in the distribution of GPs has decreased compared to the years before the HTP (the value of the index has gradually decreased except in 2016). In another study conducted in Tehran province about the distribution of human resources, GPs had the lowest Gini coefficient among healthcare workers in 2007, 2008 and 2012 [24]. In a similar study, Ghaderi et al. concluded that the HHI for GPs in Sistan and Baluchestan Province showed a moderate concentration, indicating inequality in the distribution of GPs in the province [17]. The results of this study were inconsistent with our findings (in Qazvin Province, we see a high concentration of GPs).

In urology, the HHI gradually declined, but inequality remained high. In infectious diseases, this index has not changed except in 2017, indicating an evident inequality. In general surgery, the index value is low, but inequality persists. The index value is low in anesthesia and neurology, but equality has not occurred. In pathology between 2014 and 2015, an increase in concentration was observed. Due to its allocation after the HTP in sports medicine, there is still concentration and inequality. Ghaderi et al. Showed that the HHI in specialist has increased from 0.2 in 2009 to 0.18 in 1996 [17], which is inconsistent with the results of our findings. Another study found that specialists had the highest value of the Gini coefficient among healthcare workers in Tehran province in 2010, 2011 and 2013 [25].

Allergy and immunology, vascular surgery, pediatric cardiology and pediatric urology experienced an increased concentration at the end of 2017. The concentration has been as high as in the years before the HTP in other subspecialists. No other studies focused on the distribution of these specialties.

More concentration of physicians in the centre of the province and more developed cities with better living conditions and higher income will lead to more concentration of health care workers such as physicians in these cities. They will hurt the health status of people in small cities. This issue was observed at specialized and sub-specialized levels in Qazvin. In addition, the shortage of physicians in the cities of Qazvin Province has led to sending patients to Qazvin city and increasing the indirect costs to patients’ health, which is contrary to the HTP goals. Imposing more costs will reduce patient satisfaction. A study in Guangxi in China also demonstrated that the health human resources were primarily concentrated in the more affluent populations [26].

In addition, according to HHI calculations, the number of specialists in some cities is still much lower than acceptable. Some cities even have shortcomings in basic specialists. According to the WHO report titled “health workforce requirements for universal health coverage and the sustainable development goals”, the resulting “SDG index threshold” of 4.45 doctors, nurses and midwives per 1000 population was an indicative minimum density of the need for health workers [27]. Therefore, a precise and fair estimation of the required workforce can reduce families' medical costs and improve the efficiency of health resources. However, in some cities of Qazvin Province, the lack of physical resources such as general, specialized and subspecialized hospitals may increase inequality. Another study also revealed that the level of expertise and the educational level of human resources decreased by moving from Tehran as the capital to smaller cities, such as Islamshahr [25].

The present study was not directly related to human beings. However, all ethical considerations were observed, including confidentiality, honesty in data collection, and all the authors' rights to the sources. For further research, we recommend studying the implementation barriers of the HTP Plan in the equitable distribution of health resources, investigating the reasons for reducing the physicians’ motivation to provide services to deprived areas despite deprivation coefficients applied after the HTP, evaluating the success of the HTP in equitable distribution of human resources using other indicators such as dissimilarity index, Hull Tideman, Robin Hood, etc. One reccommandation is to affect the supply of health workers via regulation of admissions in education and training programs to serve in the required regions which need more support. Other recommendations include implementing targeted recruitment for the shortages, reducing out-migration and increasing in-migration, and improving retention via contractual policies and attractive packages.

## Conclusion

According to the service dimensions of the HTP, such as ensuring fair and equitable distribution of physicians and subspecialists throughout the country and improving hoteling and renovation in the public sector, it was expected that the shortage of physicians in deprived areas would be solved. Nevertheless, our findings indicate the failure of this plan to achieve the equitable distribution of the workforce. We recommend reforming redistributive policies and paying more attention to the distribution of health resources among the cities of Qazvin province in less developed areas. Besides, adopting implementing policies to accept local residents in specialized fields could improve the status.

### Rigor of study

This is the first kind of study conducted at Qazvin University of Medical Sciences. Nonetheless, we faced some limitations. The major one was a lack of accurate information on the number of general practitioners and specialists. Researchers tried to obtain information from several sources (Deputy Minister of Curative Affairs, Iran’s Medical Council, Deputy Minister of Public Health, Hospital Information System, the Ministry of Health and Medical Education website) to solve this problem. It caused more time to get the correct information.

## Data Availability

The datasets used and/or analyzed during the current study available from the corresponding author on reasonable request. The entire dataset is in Farsi language. The Data can be available in English language for the readers and make available from the corresponding author on reasonable request.

## Abbreviations

HTTP: Health Transformation Plan
GPs: General Practitioners
